# Analysis of logistics efficiency in Guangdong and its neighboring provinces based on the DEA-Malmquist index

**DOI:** 10.1371/journal.pone.0328936

**Published:** 2025-07-24

**Authors:** Yun Wang, Huizhen Jin, Wenbin Qian

**Affiliations:** 1 School of Emergency Technology, Guangzhou Vocational College of Technology and Business, Guangzhou, Guangdong, China; 2 School of Economics and Management, Hunan University of Science and Engineering, Yongzhou, Hunan, China; Universidade de Lisboa Instituto Superior Tecnico, PORTUGAL

## Abstract

With the development of economic globalization, logistics has become a crucial link in the global supply chain. In the backdrop of growing environmental concerns and the need for more efficient business operations, sustainable logistics and advanced supply chain management have emerged as imperative components. Effective supply chain management.The region centered on Guangdong Province is one of the most active regions in the economic belt, and its geographical, industrial and cultural advantages make it one of the main centers of the national economy. This paper aims to study the regional logistics design centered on Guangdong province, with a special emphasis on incorporating sustainable logistics and supply chain management aspects. It analyzes the current situation and existing problems based on the data envelope analysis (DEA) and Malmquist index model, and conducts the logistics design analysis.The research results show that (1)The technical efficiency and technological progress of Guangdong province and its neighboring provinces have promoted the improvement of its logistics efficiency.(2)The growth of pure technical efficiency is an important factor driving the improvement of logistics efficiency in Guangdong Province and its neighboring provinces.(3)In terms of time evolution,the technological progress index had the least driving effect and should be further strengthened.(4)In 2020, due to the impact of the epidemic, the total factor productivity declined.

## 1. Introduction

### 1.1. The significance of evaluating logistics efficiency

With the vigorous development of the e–commerce network, the development of the logistics industry is playing an increasingly important role. In this context, sustainable logistics and advanced supply chain management have become key elements driving the evolution of the logistics field.Sustainable logistics focuses on reducing the environmental impact of logistics activities. This includes measures such as promoting the use of electric or hybrid vehicles in last – mile delivery to cut down on carbon emissions, implementing reusable packaging solutions to minimize waste generation, and optimizing logistics routes to reduce fuel consumption. For example, some logistics companies are now using electric tricycles for urban deliveries, which not only reduces air pollution but also noise pollution in densely populated areas.Advanced supply chain management is about integrating all aspects of the supply chain, from the procurement of raw materials to the delivery of the final product to the consumer. It emphasizes seamless information flow, efficient inventory management, and strong cooperation among all supply chain partners. In the e – commerce era, real – time data sharing between e – commerce platforms, logistics providers, and suppliers enables better demand forecasting. This helps in reducing overstocking and under – stocking situations, leading to cost savings.

### 1.2. The macro significance

At the macroeconomic level, the logistics industry, with the support of sustainable logistics and supply chain management, is the arterial system of the national economy. Sustainable logistics practices can contribute to a more environmentally friendly economic growth model. By reducing waste and emissions in the logistics process, it aligns economic development with environmental protection goals. Effective supply chain management, on the other hand, can further enhance the resource – integrating ability of the logistics industry. It closely links all links of production, circulation, and consumption, promotes the efficient circulation of goods and services, and accelerates the optimization and upgrading of the industrial structure. For instance, through better supply chain coordination, industries can respond more quickly to market changes, leading to increased productivity and a positive role in promoting GDP growth.For enterprises, good logistics, when combined with sustainable logistics and supply chain management concepts, helps enterprises to reduce costs and enhance their market competitiveness. Sustainable logistics initiatives can lower energy costs and waste – disposal costs for enterprises. Supply chain management strategies such as just – in – time inventory management can reduce inventory – holding costs. Moreover, a well – managed supply chain can improve product quality and delivery speed, which are important factors in attracting and retaining customers.For consumers, the progress of logistics, influenced by sustainable logistics and supply chain management, enables consumers to receive the purchased goods faster and have more kinds of goods to choose from. Sustainable logistics ensures that the products are delivered in an environmentally responsible way. For example, the use of biodegradable packaging materials means that consumers can enjoy their purchases without worrying about excessive environmental pollution. The seamless supply chain management also guarantees that fresh food can be delivered in a timely manner, maintaining its freshness and quality. The smooth purchase of transnational goods is also made possible by efficient supply chain operations, which involve customs clearance, international transportation, and local distribution coordination.

### 1.3. Microscopic significance

From the perspective of regional coordinated development, the logistics industry, with the support of sustainable logistics and supply chain management, can strengthen the economic links between regions. Sustainable logistics can promote the use of local, renewable resources in the logistics process, which helps local economies. Supply chain management can break through regional restrictions by integrating regional resources and promoting the division of labor and cooperation between regions. For example, regions rich in certain resources can focus on production, while other regions with better logistics infrastructure can handle distribution. This cooperation can transform the resource advantages of different regions into economic advantages and narrow the economic gap between regions.Under the tide of economic globalization and regional integration, the logistics industry of Guangdong province and its neighboring provinces is in a critical period of vigorous development. As a strong economic province in China, Guangdong’s GDP ranks among the top in China all year round, with complete industrial system and highly developed manufacturing and trade. Guangzhou’s automobile manufacturing and the electronic information industry in Shenzhen have all provided a massive supply of goods for the development of logistics. And the economic development of neighboring provinces also has its own characteristics and potential. With the developed private economy and Marine economy, Fujian has formed industrial clusters in clothing, footwear and other fields, bringing strong demand for logistics. In the process of actively undertaking industrial transfer in Jiangxi and Hunan provinces, the scale of the manufacturing industry is constantly expanding, and the featured agricultural products are rich, which also generates a large number of logistics demands. Relying on its unique geographical position, the demand for cross-border logistics continues to grow in its trade with ASEAN. The continuous improvement of transportation infrastructure has paved a solid runway for the development of the logistics industry in these areas. In Guangdong province, expressways and railways, like dense veins, run through the whole province. Guangzhou, Shenzhen and other international airport routes throughout the world, annual passenger throughput and cargo throughput amazing; Shenzhen port, Guangzhou port port container throughput among the world. Adboring provinces also spare no effort in transportation construction. Hunan has vigorously promoted the construction of the “seven vertical and seven horizontal” expressway network, and the railway network has been continuously encrypted, which has improved the convenience of cargo transportation. Guangxi has made full efforts to promote the construction of a new land-sea corridor in the western region, strengthened its land-and sea-land connection with ASEAN countries, and greatly enhanced its sea-land combined transport capacity. The development of the logistics industry is more inseparable from the strong support of policy, policy support has become a strong booster for the development of the logistics industry. The in-depth implementation of the “Belt and Road” initiative has brought great opportunities for Guangdong, Guangxi and other coastal and border provinces to deepen logistics cooperation with countries along the belt and Road. Local governments have also introduced a series of preferential policies. For example, Guangdong encourages logistics enterprises to increase investment in technological innovation and equipment upgrading through financial subsidies and tax incentives; Jiangxi provides special fund support for the construction of logistics parks to attract logistics enterprises to settle in and gather. In addition, the booming rise of the e-commerce industry has injected new vitality into the development of the logistics industry. Guangdong leads in the development of e-commerce industry, and Guangzhou, Shenzhen and other places have become important hubs of e-commerce logistics in China. The massive orders of e-commerce platforms have promoted the rapid development of express delivery and warehousing. The e-commerce industry in neighboring provinces is also developing rapidly. The retail sales of e-commerce in Fujian are increasing year by year, and many e-commerce enterprises are constantly emerging, which has driven the rapid growth of the business volume of local logistics enterprises, and also given birth to new logistics models such as direct distribution in origin and cloud warehouse, which has greatly changed the traditional logistics pattern.

### 1.4. Background of research methods

In the 1970s, operations radiologists Charnes, Cooper, and Rhodes proposed data envelope analysis methods. At that time, the traditional efficiency evaluation method was mainly measured by a single financial index or ratio, and this method has limitations.In the study of logistics efficiency, sulfur dioxide emissions, total freight volume, total import and export, financial transportation expenditure, postal business total, and number of civilian vehicles are important input-output indicators. They cannot be measured by a single index. Therefore, a method for efficiency assessment that of comprehensive evaluation of multiple input and output indicators is needed.The DEA method is able to evaluate the relative efficiency of decision units with multiple inputs and multiple outputs without predetermined the functional relationship between input and outputs. It has a wide range of applications, covering transportation, finance, medical care, education and many other fields, providing an effective tool for the evaluation of resource utilization efficiency and production performance.

Based on this,This paper is divided into six parts: introduction, literature review, method introduction, index selection, empirical analysis and conclusionto analyze the logistics efficiency of Guangdong province and its neighboring provinces.

## 2. Literature review

### 2.1. A review of domestic studies

The study of regional logistics efficiency is a hot topic among scholars.Li Minjie et al. (2025) clarified the coupling and coordinated development mechanism of digital economy and green logistics. Using methods such as the vertical and horizontal grading method, an adjusted coupling coordination degree model, hot-spot analysis, and dynamic aggregation fuzzy set qualitative comparative analysis (fsQCA), they revealed the spatiotemporal characteristics and configuration paths of the coupling and coordinated development of China’s digital economy and green logistics from 2013 to 2021. In terms of temporal changes, both the development levels of China’s digital economy and green logistics showed a fluctuating upward trend, with their coupling coordination degree also showing an increasing trend. The two have experienced a phased transition from being on the brink of imbalance to barely coordinated, but overall, the coupling coordination degree remains low [1].Wang Yanling et al. (2025) used methods such as location Gini coefficient, location entropy value, and spatial autocorrelation to conclude that the level of regional logistics industry agglomeration in China is insufficient, there are significant differences in the integration and specialization levels of the logistics industry across provinces, and regional logistics exhibits positive spatial correlation characteristics. Each region shows a development trend from dispersion to agglomeration and then back to dispersion [2]. Han Liping et al. (2024) proposed differentiated logistics industry development strategies for different provinces based on factor analysis and the coupling coordination degree model. Provinces with ultra-high scores should focus on enhancing the international competitiveness of their domestic logistics industry; high-scoring provinces should leverage emerging digital technologies to accelerate the transformation and upgrading of the logistics industry; medium-scoring provinces should promote innovation and upgrades in the logistics industry by focusing on supply chain innovation, while fostering interaction and integration between the logistics industry and new industries and business forms; low-scoring provinces need to fully tap into the policy potential of the logistics industry, strengthen logistics infrastructure construction, and expand the geographical scope and market depth of logistics services [3].Lin Xiaofang et al. (2024) used the entropy weight method to determine that high-quality development in the logistics industry has a certain spatial spillover effect. The Moran scatter plot shows that most provincial sample points are distributed in the first and third quadrants, exhibiting high-high and low-low clustering phenomena. The four major regions—eastern, central, western, and northeastern—perform differently in the four primary indicators: development foundation, development performance, innovation potential, and green low-carbon. Based on this, corresponding development recommendations are proposed [4].Wang Juan (2024) proposed that the improvement of technological progress efficiency is the main factor to promote the continuous improvement of cost reduction and efficiency in the logistics industry by constructing DEA model and Malmquist index [5].Dai Yingshuai (2024) used the value-added output of the logistics industry, direct carbon emissions from the logistics sector, and input-output models to calculate the implicit carbon emissions. The study measured the direct carbon emission levels of the logistics industry in various provinces of China and used input-output models to estimate the net carbon transfer levels of the logistics industry in each province. Based on the principle of consumption responsibility, the study further categorized the carbon emission responsibilities of the logistics industry in each province, with provinces that have net carbon transfers bearing more responsibility for carbon emissions [6].

Peng Yongfang et al. (2024) used the entropy value method to study the eastern region of China, which includes all leading provinces. The overall level of high-quality development in the logistics industry is relatively high. Most provinces in the central region are intermediate, while most provinces in the western region are lagging. The level of high-quality development in the logistics industry gradually decreases from east to west, indicating significant spatial disparities in the high-quality development of China’s logistics industry [7].Chen Xiangbing et al. (2024) used the DEA-Malmquist index model to find that the logistics efficiency in central China did not reach an effective level from 2012 to 2021, with technology being the primary issue affecting logistics efficiency across provinces in the region. Additionally, productivity showed slight decline. To address these issues, the central region needs to enhance regional collaboration and promote advancements in logistics technology. It also requires exploring innovative talent cultivation systems to attract high-tech professionals [8].Shen Haiyan (2024) used the DEA-Malmquist index model to find that from 2011 to 2021, the average growth rate of total factor productivity in the logistics industry in Beijing-Tianjin-Hebei region was 4.3%, and the change trend was fluctuating. Among them, technological progress was the main reason affecting the change of total factor productivity [9].Han Baiqing et al. (2023), based on the traditional DEA model and combined with super-efficiency DEA and Malmquist index model, showed that unreasonable resource allocation and redundant input led to the imbalance of logistics efficiency development in the region, and the comprehensive logistics efficiency of eastern coastal provinces was higher than that of central and western provinces [10].Jiang Tiejun et al. (2023) applied the output-oriented super-efficiency data envelopment analysis model to propose that performance orientation can comprehensively consider the development and changes in historical performance levels. Based on performance evaluation results, it quantifies performance targets, which is more clear and guiding compared to traditional methods due to its strong feasibility. While reinforcing the application of performance evaluation results, it also provides a new method for setting performance targets for equipment maintenance funds [11].Sun Ni (2022) et al. used DEA-Malmqusit index model to measure the efficiency of agricultural products logistics in Anhui province. The study found that the total factor productivity index of agricultural products logistics in Anhui province was in the same trend as the technological progress rate of agricultural products logistics [12].

Wei Songbo (2022) et al. used the BCC model and the Malmquist index model to analyze the overall efficiency, pure technical efficiency, and scale efficiency of the logistics industry in 10 representative cities of the “China-Europe Railway Express” hub cities. They found that the popularization of e-commerce logistics in rural areas can not only drive the development of the rural economy but also promote the overall economic level of our country [13]. Tan Tingting (2022) et al. used the DEA model and Malmquist index to analyze the changes in logistics comprehensive efficiency along the “Belt and Road” region over time and space. The study found that during the observation period, the overall logistics comprehensive efficiency of the “Belt and Road” region was relatively low. The logistics efficiency of provinces along the route generally showed a fluctuating upward trend, with faster growth in the southeastern region and slower growth in the northwestern region [14]. Liu Huihong et al. (2021) used the DEA-Malmquist total factor productivity index method to propose that the pilot cities of the Yangtze River Delta supply chain should attach importance to the application of scientific and technological innovation, increase the coordinated development among cities to expand scale benefits, and the government should guide the rational allocation of resources in an orderly manner [15]. Ding Yinan (2021) et al. used the DEA model and Malmquist index to analyze the changes in agricultural product logistics efficiency in Henan Province, thereby revealing the reasons for these changes and proposing suggestions to improve logistics efficiency. The results show that Henan Province needs to adjust its resource allocation from multiple perspectives and enhance its resource utilization capabilities; the pure technical efficiency of Henan Province exhibits characteristics of low growth rate and slow growth [16]. Li Yuqiao (2021) used the DEA-Malmquist index model to study the logistics efficiency of each province in southwest China from 2010 to 2019. The study found that the logistics development efficiency in southwest China was relatively low, and there were differences in logistics development among different provinces [17]. Li Jinhong (2021) et al. comprehensively applied the DEA model and Malmquist index to analyze the changes in the overall efficiency and total factor productivity of China’s agricultural product logistics over time and space. The results show that the overall efficiency of China’s agricultural product logistics is relatively low, with a “W” shape trend over time and a significant spatial distribution pattern that is higher in the east, lower in the middle, and lower in the west. Low pure technical efficiency and slow growth rate are key factors constraining the overall development of China’s agricultural product logistics industry [18].

Chu Yanchang (2020) et al. measured the operational efficiency of listed logistics companies in China using the DEA-Malmquist method and explored key influencing factors with the Tobit model. The study results show that the operational efficiency of listed logistics companies in China generally shows an upward trend, primarily driven by technological progress. Notably, there was a significant increase in operational efficiency between 2014 and 2015, after which it remained relatively stable [19].Yin Fengchao (2020) used the super-efficiency DEA-Malmquist model to measure and evaluate the efficiency, and measured the total factor productivity; the study found that the overall low-carbon logistics efficiency in central and western China was significantly lower than that in eastern cities [20].Gao Kang (2019) et al. used the super-efficiency DEA-ESDA method to study the spatial pattern and differences in logistics efficiency in western China; the results show that there is spatial hierarchical heterogeneity in logistics efficiency, which decreases from southwest to northwest. There is a “Matthew effect” in logistics efficiency between the two major geographic regions, with high-level efficiency converging in the southwest and low-level efficiency converging in the northwest. Moreover, the southwest region exhibits a strong “spatial lock-in effect.” [21].Zhang Xin Yue (2019) used the Malmquist index method to conduct a dynamic analysis of the development of logistics efficiency in the Beijing-Tianjin-Hebei region and evaluated the logistics efficiency in the Beijing-Tianjin-Hebei region at the same time; the research found that the logistics resources in the Beijing-Tianjin-Hebei region could not be fully utilized and the coordinated development was insufficient; the main factor affecting the change of the total efficiency in the Beijing-Tianjin-Hebei region was the technical progress index [22].Zhang Mengwu (2019) et al. applied DEA method and Malmquist total factor productivity model to comprehensively evaluate the dynamic efficiency and static efficiency of logistics industry in Shaanxi Province from 2007 to 2016; the results showed that the low pure technical efficiency and the decline of technology application level were the main reasons for the low efficiency of logistics industry in Shaanxi Province [23].Zhang Lei (2018) et al. used DEA-Malmquist index method to empirically measure the efficiency of agricultural products logistics in all leagues and cities of Inner Mongolia. The study shows that the technological progress change index of each league and city is obviously less than 1, and the efficiency decreases significantly [24].

Yu Liying (2018) et al. used DEA and Malmquist index methods to conduct a dynamic analysis of the development of logistics efficiency in the Yangtze River Economic Belt from changes in total efficiency, technical efficiency, and technological progress. They also drew spatial distribution maps to reflect the characteristics of changes in logistics efficiency. The study found that the development of the logistics industry in the upper and middle reaches of the Yangtze River Economic Belt is relatively unbalanced. In contrast, while the downstream regions have better logistics efficiency, their growth rate has slowed down. The logistics efficiency in the upper and middle reaches lags behind but has significant room for improvement. Overall, technological progress is a crucial factor influencing changes in total efficiency [25].Cheng Shuqiang (2017) et al. explored the changes in agricultural product logistics efficiency, inter-provincial differences, and their causes using the DEA-Malmquist index method; the study found that the efficiency of agricultural product logistics in western regions has been increasing year by year but at a slower pace, with technological progress being the primary driver of efficiency improvement, while technical and scale efficiency have played a hindering role; the differences among provinces and cities have narrowed over the years, with the level of technology application being the main reason for these differences; the impact of industrial structure on efficiency varies at different stages [26].

### 2.2. A review of foreign studies

ZhiyongZeng (2023) puts forward four aspects to improve the service quality of express delivery enterprises: expanding network coverage, establishing and improving the picking mechanism, scientific classification management of express delivery, and enhancing employees’ sense of identity with the enterprise [27].LiliZhu (2023) used methods such as literature review, data analysis, and comparative analysis to examine the scale of cross-border e-commerce trade, product structure, and market structure in Guangdong Province. The study identified four main issues: weak brand awareness among enterprises; a shortage of specialized talent for cross-border e-commerce; an underdeveloped logistics system with high costs; and restricted customs clearance conditions and the need for improved payment credit. Based on these issues, the study proposed four improvement measures: fostering brand awareness and strengthening brand building; enhancing the introduction and training of cross-border e-commerce merchants; improving infrastructure to optimize logistics costs; and increasing customs clearance efficiency and perfecting the construction of cross-border payment platforms, thereby promoting better development of cross-border e-commerce in Guangdong Province [28], see Table 1.

**Table1:** Summary of key studies on logistics efffciency.

Author (s)	Study	Methodology	Methodology
Li Minjie (2025)	Mechanism for coordinated development of digital economy and green logistics	Summarized fuzzy set qualitative comparative analysis (fsQCA)	The development level of digital economy and green logistics in China is on the rise with fluctuations
Peng Yongfang (2024)	Eastern China	entropy evaluation method	There are significant spatial differences in the high-quality development of logistics industry in China
Chen Xiangbing (2024)	Logistics efficiency in central China	DEA-Malmquist	Technology is the main problem of logistics efficiency in central provinces, while productivity has also declined slightly
Shen Haiyan (2024)	Logistics industry in the Beijing-Tianjin-Hebei region	DEA-Malmquist	The trend of total factor productivity in the logistics industry in the Beijing-Tianjin-Hebei region is fluctuating, and technological progress is the main reason affecting the change of total factor productivity
Sunnie (2022)	Agricultural products logistics efficiency in Anhui	DEA-Malmqusit	The total factor productivity index of agricultural products logistics in Anhui province is the same as the change trend of technological progress rate of agricultural products logistics
Wei Songbo (2022)	Hub city of China-Europe freight trains	BCC&Malmquist	The popularization of e-commerce logistics in rural areas can not only promote the development of rural economy, but also promote the development of China’s overall economic level
Tan Tingting (2022)	The comprehensive efficiency of logistics along the “Belt and Road” route	DEA-Malmquist	The overall logistics efficiency of the “Belt and Road” region is relatively low, and the logistics efficiency of all provinces along the “Belt and Road” shows a fluctuating upward trend, with the southeast region growing faster and the northwest region growing slower.
Ding Yinan (2021)	Logistics efficiency of agricultural products in Henan Province	DEA-Malmquist	The pure technical efficiency of Henan province shows the characteristics of low growth rate and slow growth
Li Yuqiao (2021)	Logistics efficiency of various provinces in southwest China	DEA-Malmquist	The development efficiency of logistics in southwest China is low, and there are differences in the development of logistics among different provinces
Li Jinhong (2021)	Comprehensive efficiency of agricultural products logistics in China	DEA-Malmquist	The overall efficiency of China’s agricultural products logistics is low, and the low pure technical efficiency and slow growth rate are the key factors restricting the overall development of China’s agricultural products logistics industry
Chu Yan-chang (2020)	Operating efficiency of listed logistics enterprises in China	DEA-Malmquist&Tobit	The operation efficiency of listed logistics enterprises in China is on the rise, and its change is mainly determined by technological progress
Yin Fengchao (2020)	Low carbon logistics efficiency in central and western China	Super efficiency DEA-Malmquist	The overall efficiency of low-carbon logistics in the central and western regions is significantly lower than that in the eastern cities
Gao Kang (2019)	Spatial pattern and differentiation of logistics efficiency in western China	Super efficiency DEA-ESDA	Logistics efficiency has spatial hierarchical heterogeneity and shows a decreasing trend from southwest to northwest
Zhang Xinyue (2019)	Logistics efficiency in the Beijing-Tianjin-Hebei region	Malmquist	The logistics resources in the Beijing-Tianjin-Hebei region cannot be fully utilized and the coordinated development is insufficient; the main factor affecting the change of the total efficiency of the Beijing-Tianjin-Hebei region is the index of technological progress
Zhang Mengwu (2019)	Logistics industry in Shaanxi Province	DEA-Malmquist	The low level of pure technical efficiency and the decline of technology application are the main reasons for the low efficiency of logistics industry in Shaanxi Province
Lei Zhang (2018)	Logistics efficiency of agricultural products in Inner Mongolia	DEA-Malmquist	The index of technological progress change in each alliance city is obviously less than 1, and the efficiency decreases significantly
Yu Liying (2018)	Logistics efficiency of the Yangtze River Economic Belt	DEA-Malmquist	The development of logistics industry in the upper and middle reaches of the Yangtze River Economic Belt is not balanced, and technological progress is an important factor affecting the change of total efficiency
Cheng Shuqiang (2017)	Agricultural product logistics efficiency in western China	DEA-Malmquist	The efficiency of agricultural products logistics in western China is increasing year by year, but the growth rate is slowing down
Gan Weihua (2015)	Total factor productivity of logistics industry in Jiangxi Province	DEA-Malmquist	The key to the growth of Malmquist index in logistics industry in various cities of Jiangxi province lies in technological progress

## 3. Method introduction

### 3.1. Data envelope analysis

Data envelope analysis (DEA) is a linear program to evaluate the effectiveness of multiple decision units, and is a non-parametric method for comprehensive evaluation and analysis. In order to study the logistics efficiency of Guangdong province and its adjacent provinces, the ultra-efficiency model optimization DEA will be calculated. Among them, the total import and export volume, the total freight volume and sulfur dioxide were the explained variables, and the explanatory variables with other influencing factors were used for constructing the final regression model, and stata software was used for data analysis. Suppose there are n DMUs (decision units), each DMU has m inputs and s output, DUM_j_ (j = 1,..., n) is j, its i input is x_ij_ (i = 1,..., m), its r output is y_rj_ (r = 1,..., s), and the evaluated DMU is recorded as DUM_p_ (p∈ {1,..., n}). Where (g_i_^x^,g_r_^y^) represents the direction vector, satisfying g_i_^x^,g_r_^y^ ≥ 0,∀i, r, and (g_i_^x^,g_r_^y^) ≠0. The specific formula is as follows:


$$\sum_{j=1,j\neq p}^{n}\lambda_{j}=1,\lambda_{j}\geq 0,j=1,...,n,\;j\neq p$$ (1)

$$\begin{array}{cc} & \max\beta_{p}\\ & s.t.\left\{ \begin{array}{c} @l\sum_{j=1,j\neq p}^{n}\lambda_{j}x_{ij}\leq x_{ip}-\beta_{p}g_{i}^{x},i=1,...,m.\\ \sum_{j=1,j\neq p}^{n}\lambda_{j}y_{rj}\geq y_{rp}+\beta_{p}g_{r}^{y},r=1,...,s.\\ \sum_{j=1,j\neq p}^{n}\lambda_{j}=1,\lambda_{j}\geq 0,j=1,...,n. \end{array}\right. \end{array}$$ (2)

The optimal value of the model is β_p_^*^, and β_p_^*^ measures the invalidation of the evaluated unit (DUM_p_). If β_p_^*^ > 0 indicates that when the input is compressed proportionally, the corresponding output should expand proportionally; if β_p_^*^ < 0, if the input is expanded proportionally, the corresponding output should be compressed proportionally. A smaller p indicates the more effective DUM evaluated and the β_p_^*^ ≤ 0 of the effective DUM. The DUM super-efficiency value can be defined as:1-β_p_^*^.

### 3.2. Malmquist The exponential model

The Malmquist exponential model was originally proposed by Malmquist in 1953, and Caves, Christensen, and Diewert began to apply this exponential model to measure production efficiency changes in 1982. After 1994, the Malmquist index was widely used. Nowadays, this method is widely used in the measurement of production efficiency, and the international comparison is based on the results of efficiency measurement.Due to the limitations of static DEA, we use DEA combined with Malmquist index model for dynamic analysis.The Malmquist index represents the dynamic change process of each decision unit from t to t + 1 efficiency value (DMU), with x_t_ as the input variable and y_t_ as the output variable. The tfpch is the total factor productivity change index, which refers to the comprehensive productivity of each production factor of the production unit in the system; effch is the technical efficiency change index, which refers to the technological improvement and innovation to improve product quality and production capacity; techch is the technological progress change index, aiming to evaluate whether the resources are fully utilized in the production process. When Tfpch = 1, it means that the influence of each factor on production efficiency remains unchanged; when Tfpch >1, it means that each factor contributes more to production efficiency; when Tfpch<1, each factor contributes less to production efficiency.

When the return of scale is unchanged, there is the following formula:


$$\begin{array}{cc} & Tfpch=effch\times techch\\ & ={\left(\frac{D_{0}^{t}\left(x_{t+1},y_{t+1}\right)}{D_{0}^{t}\left(x_{t},y_{t}\right)}\times \frac{D_{0}^{t+1}\left(x_{t+1},y_{t+1}\right)}{D_{0}^{t+1}\left(x_{t},y_{t}\right)}\right)}^{\frac{1}{2}}\\ & =\frac{D_{0}^{t+1}\left(x_{t+1},y_{t+1}\right)}{D_{0}^{t}\left(x_{t},y_{t}\right)}{\left(\frac{D_{0}^{t}\left(x_{t+1},y_{t+1}\right)}{D_{0}^{t+1}\left(x_{t+1},y_{t+1}\right)}\times \frac{D_{0}^{t}\left(x_{t},y_{t}\right)}{D_{0}^{t+1}\left(x_{t},y_{t}\right)}\right)}^{\frac{1}{2}} \end{array}$$ (3)

When the scale return is variable, the technical efficiency change index can be further disassembled into the pure technical efficiency change index (pech) and the scale efficiency change index (sech). The former measures whether the factors of production have exerted their production potential, while the latter measures whether production and operation at an appropriate scale. The decomposition formula is as follows:


$$\begin{array}{cc} & Effch=pech\times \mathrm{sech}\\ & =\frac{D_{0}^{t+1}\left(x_{t+1},y_{t+1}\right)}{D_{0}^{t}\left(x_{t},y_{t}\right)}\\ & =\frac{D_{0}^{t+1}\left(x_{t+1},y_{t+1}\right)}{D_{0}^{t}\left(x_{t},y_{t}\right)}\left(\frac{D_{0}^{t+1}\left(x_{t+1},y_{t+1}\right)/D_{0}^{t+1}\left(x_{t+1},y_{t+1}\right)}{D_{0}^{t}\left(x_{t},y_{t}\right)/D_{0}^{t+1}\left(x_{t+1},y_{t+1}\right)}\right) \end{array}$$ (4)

## 4. Empirical analysis

### 4.1. Index selection

As shown in Table 2, the number of civil vehicles is the basis of highway logistics transportation and the reflection of highway transportation volume. The total amount of postal business can reflect the scale of small items, express delivery business and transportation needs. Transportation fiscal expenditure refers to the improvement of transportation infrastructure, such as highway and railway construction, improving the efficiency of logistics and transportation, and reducing costs. Therefore, these three indicators are taken as the input indicators. The total import and export volume reflects the scale of international logistics demand. Total freight volume directly reflects the business scale and activity of the logistics industry. Sulfur dioxide emission, as an unexpected output, reflects the environmental protection degree of logistics from the side, with low emission, indicating that logistics transportation adopts more environmentally friendly technology and equipment. Therefore, these three indicators are used as the output indicators. Since there are still missing data in 2023 and 2024, we selected the data from 2018 to 2022, and relying on these index data, this paper studies the logistics efficiency on Guangdong and its neighboring provinces.The data used in this study comes from the Chinese government website, China Liaison Office, State Post Bureau, People’s Daily Online, National Bureau of Statistics, provincial finance departments and wind financial terminal.

**Table 2:** Table of input-output variables.

Indicator type	index	variable	unit
Investment index	Number of civil vehicles	X1	Vehicles
	Total postal business	X2	100 million
	Transportation fiscal expenditure	X3	100 million
Output indicators	total export-import volume	Y1	100 million
	Total freight volume	Y2	Ten thousand tons
	Sulfur dioxide emissions	Undesired output	Ten thousand tons

### 4.2. Analysis of DEA-Malmquist index and decomposition index of logistics efficiency in each province

This paper uses MAXDEA8.0 software to calculate the logistics efficiency of DEA-Malmquist index and its decomposition value of Guangdong Province and its surrounding provinces. The calculation results are shown in Table 2.

As can be seen from Table 3, the overall logistics operation performance of Guangdong province and its neighboring provinces has improved, and the average total factor productivity index is 1.0429, indicating an average annual growth rate of 4.29%. Among the five provinces, only one of the total factor productivity index decreased in Jiangxi Province, which decreased by 3.325% during the inspection period, indicating that the logistics efficiency during the inspection period needs to be further improved. While the rest of the provinces are rising, Fujian province and Guangdong province total factor productivity index of 1.1195 and 1.11725, shows that the two provinces of total factor productivity index during the inspection rose 11.95% and 11.725%, respectively, the larger, but also means that the two provinces is all the research samples of the best logistics efficiency.

**Table 3:** The exponential decomposition table of DEA-Malmquist of the logistics efficiency in each province.

province	tfpch	tech	effch	pech	sech
Fujian	1.1195	1.042	1.069	1.075	1.02325
Guangdong	1.11725	1.11725	1	1	1
Guangxi	1.001	1.001	1	1	1
Hunan	1.01	0.9085	1.10025	1.098	1.0005
Jiangxi	0.96675	0.94625	1.0125	0.99575	1.01775
mean	1.0429	1.003	1.03635	1.03375	1.0083

The total factor productivity index is further decomposed into technical efficiency change index (effch) and technical progress change index (tech), and it can be found that the average value of the overall technical efficiency change index is 1.03635, indicating that the overall technical efficiency improves by a small margin of 3.635%. Among them,Hunan province has the highest technical efficiency index of 1.10025, with an average annual growth rate of 10.025%. Although the technological progress change index is low, its total factor productivity index is still greater than 1 and in an upward state, indicating that technical efficiency is the main factor driving the improvement of logistics efficiency in Hunan Province. The lowest technical efficiency index is in Guangdong province and Guangxi provinces, and the technical efficiency index of both is 1, indicating that these two provinces have full use of technology and efficient resource allocation efficiency. Research object of overall technological progress change index mean 1.003, including only the technology progress index of Hunan province and Jiangxi province is less than 1,0.9085 and 0.94625 respectively, fell 9.15% and 5.375%, due to the technical efficiency of Hunan province, total factor productivity present progress, but in Guangxi province because its technological progress index is too low, led to the total factor productivity index, it shows that technological progress is the main factor hindering logistics efficiency in Guangxi province. On the whole, the technical efficiency and technological progress of Guangdong province and its neighboring provinces have promoted the improvement of its logistics efficiency, and there is great room for improvement in team management, resource allocation and technological innovation.

In addition, except for Guangdong province and Guangxi Province, the technological efficiency change index of the other sample provinces is greater than the change index of technological progress, indicating that technological progress has a greater obstacle to the overall logistics efficiency of Guangdong province and its neighboring provinces and has more room for improvement. In the future, the development of Guangdong Province and its adjacent areas needs to continuously adjust and improve technological innovation while paying attention to enterprise management and resource allocation.

Further decompose the technical efficiency change index into the pure technical efficiency change index (pech) and the scale efficiency change index (see), it can be seen that the average value of the pure technical efficiency change index is 1.03375, which is in an upward trend, while the scale efficiency change index is 1.0083, which is also in an upward trend, but the increase is only 0.83%. This shows that on the whole, the growth of pure technical efficiency is an important factor driving the improvement of logistics efficiency in Guangdong Province and its neighboring provinces. Scale efficiency also plays a role in promoting technical efficiency changes, but the effect is not significant. By observing the index value of each province, we can find that Hunan Province has the highest pure technical efficiency index of 1.098, an increase of 9.8%, indicating that the management level of logistics in Hunan Province has improved; the highest scale efficiency index is 1.02325, an increase of 2.325%, indicating that the expansion of logistics scale in Fujian Province has brought positive economic impact. However, the overall total factor productivity of Hunan still lags behind Fujian, indicating that Hunan province still has a lot of room for progress in logistics scale.

### 4.3. Analysis of the annual logistics efficiency DEA-Malmquist index and decomposition index of each province

Malmquist It can dynamically reflect the changes of the overall logistics efficiency of Guangdong Province and its neighboring provinces. The DEA-Malmquist index is applied to decompose the logistics efficiency of Guangdong Province and its neighboring provinces in 2018–2022. The calculation results are shown in Table 4.

**Table 4:** The exponential decomposition table of DEA-Malmquist of the annual logistics efficiency.

	tfpch	tech	effch	pech	sech
2018-2019	0.7622	0.8134	0.9436	0.915	1.0334
2019-2020	1.2016	1.1416	1.0526	1.1244	0.9476
2020-2021	1.2856	1.1418	1.1412	1.0818	1.058
2021-2022	0.9222	0.9152	1.008	1.0138	0.9942
mean	1.0429	1.003	1.03635	1.03375	1.0083

As can be seen from Table 4: from the perspective of overall efficiency changes, the average total factor productivity index (tfp) of Guangdong province and its neighboring provinces from 2018−2022 was 1.0429, with an average annual growth rate of 4.29%. Among them, in 2018−2019 and 2021−2011,23.38% and 7.78% retreated, respectively. From 2018 to 2019, the logistics business in Guangdong province is due to the decline of the traditional market and the difficult operation. The neighboring provinces are due to the restriction of economic development level, imperfect logistics infrastructure, low level of information technology and shortage of talents. From 2021 to 2022, this period is still in the period of epidemic control. The severe impact of the epidemic on various economies has caused the difficult growth of the logistics industry, and it is difficult to achieve progress. However, during the period from 2019 to 2021, the total factor productivity index (tfp) of Guangdong province and its neighboring provinces increased, increasing by 20.16% and 28.56%, respectively, due to the growth of online consumer demand, strong policy support, logistics model innovation and technological innovation during the epidemic period.

The total factor productivity change index is further decomposed into technical efficiency change index (effch) and technological progress change index (tech), it can be found that the average technological efficiency change index (effch) is 1.03635,30 with a mean annual increase of 3.635%, while the average technological progress index (tech) is 1.003, an annual increase of 0.3%, which can be understood that the change of technological efficiency can promote the logistics efficiency of Guangdong Province and its neighboring provinces; Although the change of technological progress promotes the logistics efficiency of Guangdong Province and its neighboring provinces, this promotion effect is relatively small. During the epidemic period, it was more difficult to guarantee the basic business, and it was more difficult for enterprises to have surplus funds to invest in technological innovation. Then technical efficiency change index decomposition into pure technical efficiency change index (pech) and scale efficiency change index (see), you can see the inspection period pure technical efficiency change index mean 1.03375, an average annual growth of 3.375%, scale efficiency average of 1.0083, up 0.83%, which shows that overall pure technical efficiency and scale efficiency changes to promote the development of technical efficiency, but the role of pure technical efficiency is greater than the scale efficiency. In 2021–2022, the scale efficiency index was less than 1, indicating that the scale efficiency played a reverse role on the technical efficiency in this year.

**Fig 1:**
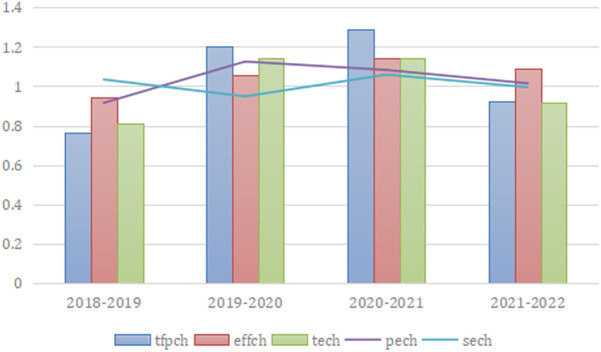
Column-line chart of five efficiency values in provinces from 2018 to 2022.

On the basis of Table 3, the Malmquist index of logistics efficiency and the decomposed time dynamic column-line chart of Guangdong Province and its neighboring provinces can be drawn to observe the changes of each index more clearly, as shown in Fig 1. From 2018 to 2020, the overall total factor productivity of the logistics industry in Guangdong province and its neighboring provinces will gradually increase gradually, and the logistics efficiency will gradually improve.However, due to the impact of the epidemic, the total factor productivity of Guangdong province will decline from 2020 to 2022. Decomposed, technological progress and technical efficiency will gradually improve from 2018 to 2020, and except for 2019–2020 effch was less than tech, 2020 and tech in 2020–2021, effch was greater than tech. Further observe the pure technical efficiency and scale efficiency, It can be found that these two efficiency indices did not change steadily during the visit, Pure technical efficiency shows a changing trend of rising first and then decreasing, While the scale efficiency shows a changing trend of rise-decline-rise, In years 2019–2020, Both fluctuate at opposite levels, There is a large gap; Between 2020–2022, It shows that the impact of the epidemic has little impact on the organization, management and resource allocation of the logistics industry in Guangdong province and its neighboring provinces, And the impact on the scale and efficiency that has already been formed changes greatly, The recovery and improvement of operational efficiency in the future need to focus on the development of enterprise scale.

## 5. Conclusion and policy recommendations

### 5.1. Conclusion

This paper makes a brief analysis of the logistics efficiency based on the relevant indicators of Guangdong province and its neighboring provinces in the five years from 2018 to 2022. After constructing the DEA-Malmquist index model, we draw the following conclusions based on the empirical analysis results:

(1) The average change index of the overall technical efficiency was 1.03635, indicating that the overall technical efficiency increased by 3.635%. On the whole, the technical efficiency and technological progress of Guangdong province and its neighboring provinces have promoted the improvement of its logistics efficiency, and there is great room for improvement in team management, resource allocation and technological innovation.
(2) The average pure technical efficiency change index is 1.03375, which is in an upward trend, while the scale efficiency change index is 1.0083, which is also in an upward trend, but the increase rate is only 0.83%. This shows that on the whole, the growth of pure technical efficiency is an important factor driving the improvement of logistics efficiency in Guangdong Province and its neighboring provinces.
(3) In terms of time evolution, from the perspective of overall efficiency changes, the average total factor productivity index (tfp) of Guangdong province and its neighboring provinces from 2018−2022 was 1.0429, with an average annual growth rate of 4.29%However, there was a decline in the periods 2018−2019 and 2021−2011. Overall, the technological progress index had the least driving effect and should be further strengthened.
(4) From the perspective of spatial evolution, from 2018 to 2020, the overall total factor productivity of the logistics industry in Guangdong province and its neighboring provinces increased slowly, and the logistics efficiency gradually improved. However, in 2020, due to the impact of the epidemic, the total factor productivity declined. Further observe pure technical efficiency and scale efficiency, explain the impact of the outbreak of Guangdong province and the allocation of resources, and has formed the scale of efficiency change is bigger, the recovery of operational efficiency and improve the future need to focus on the development of logistics scale.

### 5.2. Policy proposal

(1) Continue to promote integrated development:In the context of sustainable logistics and supply chain management, accelerate the “two advances, one out” project. When strengthening the construction of the international delivery logistics system, pay attention to optimizing the supply chain layout. This includes integrating different logistics links such as warehousing, transportation, and distribution to improve overall efficiency.
(2) Improve the supply system:Incorporating supply chain management concepts, promote the improvement of delivery service quality. In optimizing express delivery service channels, use data driven supply chain analytics to identify bottlenecks in the delivery process and streamline operations. For the improvement of the rural logistics network node system and the joint promotion of the construction of three level logistics nodes in counties, towns and villages, build a unified supply chain information platform. This platform can enable seamless information sharing among different nodes, improving the overall efficiency of rural logistics. In deepening the integrated development of rural passenger, goods and postal services, and coordinating the construction of various terminal facilities, sustainable logistics can be emphasized.
(3) Strengthen scientific and technological innovation:In actively promoting the construction of smart postal services and accelerating the construction of smart infrastructure and information infrastructure of postal big data centers, these technological advancements can be well integrated with sustainable logistics and supply chain management.In supply chain management, the smart infrastructure can enhance the traceability of goods throughout the supply chain, from the source of production to the end consumer.
(4) Promote green, low carbon and sustainable development:In innovating logistics service mode, supply chain management plays a crucial role.Strengthen the management of excessive packaging of mail express by collaborating with suppliers and e commerce platforms through supply chain cooperation. Improve the level of green, reduction, standardization and recycling of express packaging and logistics equipment. In promoting the low carbon construction and operation of facilities and places, and building green outlets and distribution centers, use sustainable construction materials and energy saving technologies.
(5) Enhance the level of opening up and cooperation:When accelerating the construction of land delivery channels connecting China and countries along the Belt and Road routes, promoting the development of international freight trains, and expanding the international aviation delivery network, sustainable logistics and supply chain management should be considered. For international freight trains, optimize the loading and unloading processes to improve the utilization rate of train carriages, reducing energy consumption per unit of transported goods. In the international aviation delivery network, cooperate with airlines to promote the use of more fuel efficient aircraft models through supply chain wide cooperation.
(6) Protecting the legitimate rights and interests of couriers:In the context of supply chain management, couriers are an important part of the last mile delivery link. Improving the mechanism for protecting the legitimate rights and interests of couriers, improving the career development guarantee system, and enhancing their sense of professional identity, sense of belonging and labor happiness can contribute to a more stable and efficient last mile delivery service in the supply chain. A happy and motivated workforce is more likely to provide high – quality service, which is beneficial for the overall development of the logistics and supply chain.
(7) Strengthen industry safety management:Continue to improve the supervision mechanism. In the supply chain, safety management should cover all aspects, from the safety of goods during transportation.
